# *In Vitro* Assessment of CYP-Mediated Drug Interactions for Kinsenoside, an Antihyperlipidemic Candidate

**DOI:** 10.3390/molecules21060800

**Published:** 2016-06-18

**Authors:** Shaheed Ur Rehman, Min Sun Choi, In Sook Kim, Zengwei Luo, Yongbo Xue, Guangming Yao, Yonghui Zhang, Hye Hyun Yoo

**Affiliations:** 1 Institute of Pharmaceutical Science and Technology and College of Pharmacy, Hanyang University, Ansan Gyeonggi-do 426-791, Korea; dr.shaheedmarwat@yahoo.com (S.U.R.); chm2456@hanyang.ac.kr (M.S.C.); kis@hanyang.ac.kr (I.S.K.); 2 School of Pharmacy, Tongji Medical College of Huazhong University of Science and Technology, Wuhan 430030, China; luozengwei@hust.edu.cn (Z.L.); yongboxue@mail.hust.edu.cn (Y.X.); gyap@hust.edu.cn (G.Y.); zhangyh@mails.tjmu.edu.cn (Y.Z.)

**Keywords:** kinsenoside, human liver microsomes, drug interactions, CYP inhibition

## Abstract

Kinsenoside, the herb-derived medicine isolated from the plant *Anoect chilus*, has diverse pharmacological actions, and it is considered to be a promising antihyperlipidemic drug candidate. This study evaluates the effects of kinsenoside on CYP enzyme-mediated drug metabolism in order to predict the potential for kinsenoside-drug interactions. Kinsenoside was tested at different concentrations of 0.1, 0.3, 1, 3, 10, 30, and 100 µM in human liver microsomes. The c Cktail probe assay based on liquid chromatography-tandem mass spectrometry was conducted to measure the CYP inhibitory effect of kinsenoside. Subsequently, the metabolism profiles of amlodipine and lovastatin in human liver microsomes were analyzed following co-incubation with kinsenoside. The concentration levels of the parent drug and the major metabolites were compared with the kinsenoside-cotreated samples. The effect of kinsenoside was negligible on the enzyme activity of all the CYP isozymes tested even though CYP2A6 was slightly inhibited at higher concentrations. The drug-drug interaction assay also showed that the concomitant use of kinsenoside has a non-significant effect on the concentration of lovastatin or amlodipine, and their major metabolites. So, it was concluded that there is almost no risk of drug interaction between kinsenoside and CYP drug substrates via CYP inhibition.

## 1. Introduction

Kinsenoside (3-*R*-3-β-d-glucopyranosyloxybutanolide) is the principal bioactive constituent of *Anoect chilus formosanus*, an important ethnomedicinal plant in Asian countries, primarily Taiwan and China [1,2,3]. Studies have reported that kinsenoside exhibits a variety of pharmacological actions, including antihyperlipidemic, antihyperglycemic, anti-inflammatory, antioxidant, immune-stimulating, anti-osteoporosis, hepatoprotective, and vasculoprotective effects [1,2,3,4,5,6,7,8,9]. All of these pharmacological activities and drug-likeness make kinsenoside a promising drug candidate, and further investigation is needed to test its efficacy and safety.

Important medicinal plants and their bioactive constituents have been used therapeutically all around the world [10]. Herbal medicines and herb-derived medicines are gaining importance very quickly because they are derived from nature; thus, they are perceived to be free of side effects [11]. However, their co-administration with conventional medicines can have life-threatening consequences [12]. For new drug entities, it is essential to determine the pharmacokinetics and the drug’s cytochrome P450 (CYP) enzyme activity and metabolic profile in order to predict their possible interactions with other drugs. CYP is a superfamily of hemoproteins that plays a central role in the oxidative metabolism (phase I, biotransformation) of xenobiotics and endogenous compounds [13,14]. In humans and in animals, CYPs can be found virtually in all body organs, predominantly in the liver and intestinal epithelia, which play a major role in CYP-mediated drug metabolism. Other tissues, including the skin, the nasal epithelia, the lungs, and the kidneys, as well as the testes, brain, and other organs, play a much smaller role in drug metabolism [15,16]. The most important CYP subfamilies are 1A2, 2A6, 2C8, 2C9, 2C19, 2D6, and 3A4; these are responsible for drug metabolism in humans [17,18]. Any combination of two or more drugs might be substrates of the same CYP isoenzyme when ingested concomitantly; this could result in induction or inhibition of the CYP isoenzyme, resulting in clinically significant drug-drug interactions that can cause unanticipated adverse reactions or therapeutic failures [19,20,21].

Hypercholesterolemia is a well-established risk factor for arteriosclerosis, ultimately leading to cardiovascular disease (CVD) and stroke, both of which are major causes of death in developed countries [22]. Clinical evidence has shown that employing a combined/multifactorial approach that uses a lipid-lowering agent with an antihypertensive agent to reduce the risk of CVD has advantages over the older sequential monotherapy approach [23,24,25,26]. The combination of amlodipine and lovastatin is primarily recommended to treat hypercholesterolemia, which can cause CVD and stroke. So, kinsenoside, a promising antihyperlipidemic drug candidate [2], could possibly be used concomitantly with these two classes of drugs.

Kinsenoside, which is an herb-derived medicine considered a promising new drug candidate, should be investigated for the inhibitory effects on CYP enzyme activities in order to predict its possible interaction with CYP drug substrates via CYP inhibition. No previous study has shown the kinsenoside CYP activity. Because pharmacological studies have revealed that kinsenoside is a promising antihyperlipidemic drug candidate, it should be directly evaluated for CYP-mediated drug interactions towards antihyperlipidemic and antihypertensive classes of drugs. Therefore, this study, based on liquid chromatography/tandem mass spectrometry (LC/MS/MS), examined the inhibitory effects of kinsenoside on the CYP450-mediated drug metabolism in human liver microsomes, and further investigated the qualitative and quantitative profile of CYP-mediated metabolites of commonly used antihyperlipidemic and antihypertensive drugs, lovastatin and amlodipine, respectively, following the treatment with kinsenoside.

## 2. Results

### 2.1. CYP Inhibition Assay

**Figure 1:**
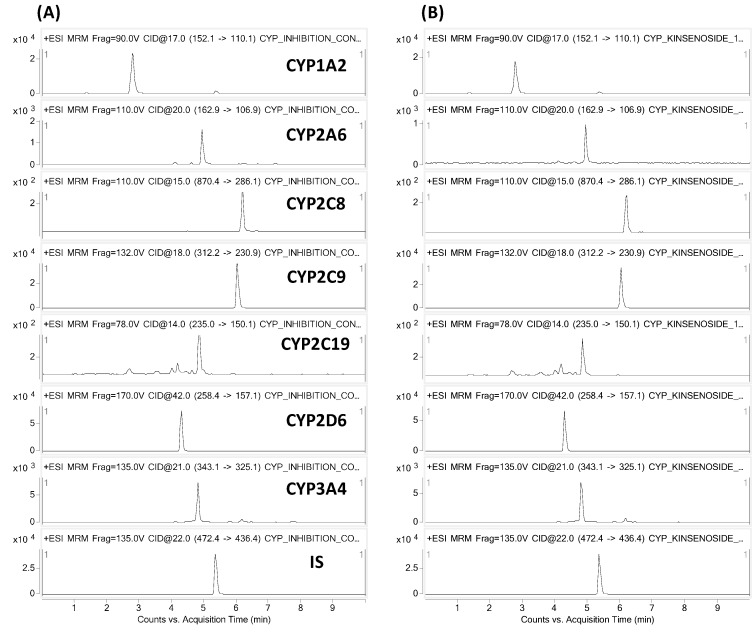
Representative MRM chromatograms of human liver microsome samples of (**A**) control and (**B**) kinsenoside (100 µM)-treated. Human liver microsomal fraction was incubated with the substrate mixture, NADPH-generating system, and kinsenoside for 30 min and the formation of the CYP-specific metabolites was determined by LC-MS/MS.

**Figure 2:**
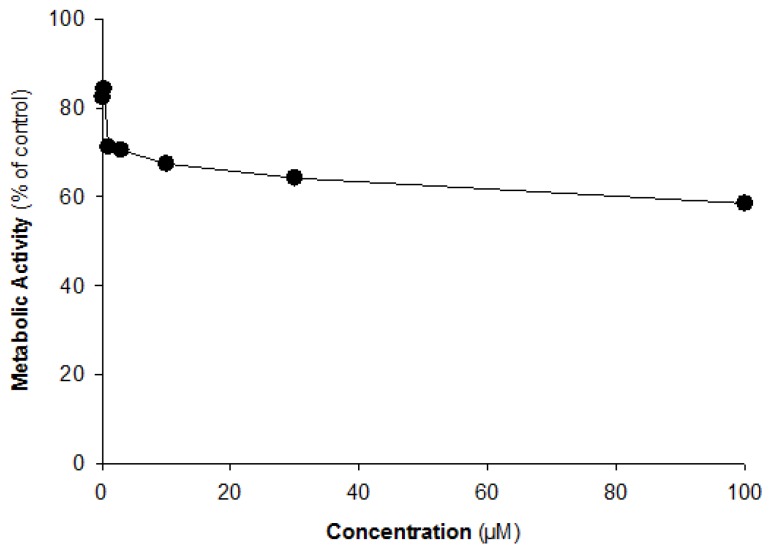
The inhibitory effects of kinsenoside on CYP2A6 enzyme activity in c-DNA expressed CYP2A6 supersomes.

The inhibitory effects of kinsenoside on CYP enzymes were investigated in human liver microsomes. The CYP inhibition assay was conducted with well-known CYP selective inhibitors. Furafylline, methoxsalen, quercetin, sulfaphenazole, ticlopidine, quinidine, and ketoconazole were used as positive controls for CYP1A2, CYP2A6, CYP2C8, CYP2C9, CYP2C19, CYP2D6, and CYP3A4, respectively. Each CYP-speciflc metabolite that was formed was reduced by >95% after applying its corresponding inhibitor, indicating that the assay system was functioning well. The inhibitory effect of kinsenoside on the metabolite formation of CYP specific substrates was investigated at concentrations of 0.1, 0.3, 1, 3, 10, 30, and 100 µM. The effects on the metabolite formation are presented as % of control (Table 1). Figure 1 shows the representative MRM chromatograms of the control and kinsenoside-treated human liver microsome samples. The results indicated that kinsenoside has a negligible inhibitory effect on six CYP isozymes, while CYP2A6-specific metabolite formation (*i.e.*, 7-OH-coumarin) was slightly inhibited in a concentration-dependent manner. To confirm the inhibition of CYP2A6 by kinsenoside, we further investigated with cDNA-expressed recombinant CYP2A6 isozyme. Kinsenoside also exhibited a slight inhibitory effect on recombinant CYP2A6 isozyme. However, the effect was shown to reach saturation at higher concentrations and remained inhibited as approximately 60% of control even at a concentration of 100 µM (Figure 2).

### 2.2. Effects of Kinsenoside on the Metabolism of Amlodipine and Lovastatin

**Figure 3:**
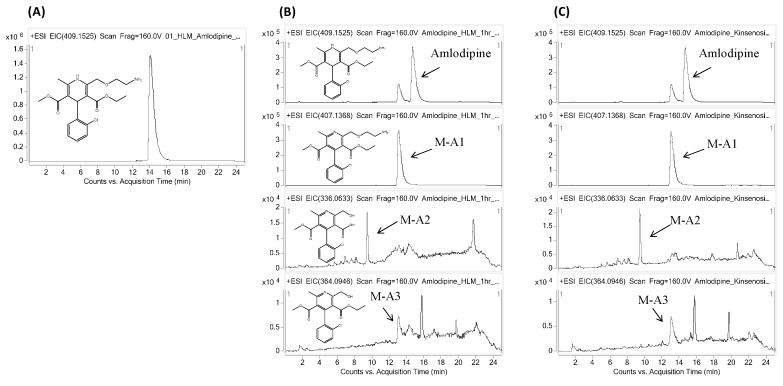
Representative EIC chromatograms for (**A**) amlodipine standard, and in human liver microsome samples (**B**) without kinsenoside (control) and (**C**) with kinsenoside.

**Figure 4:**
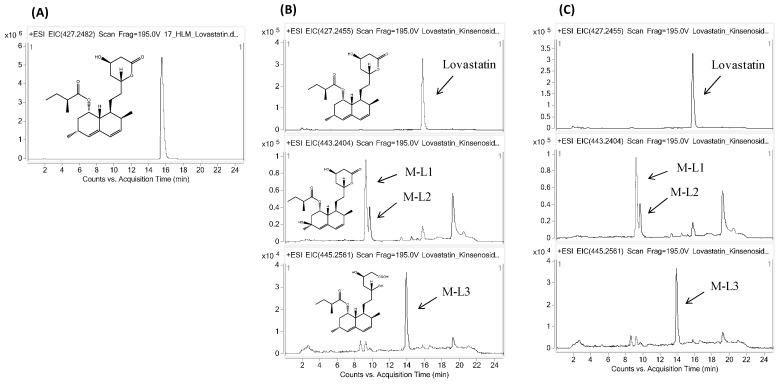
Representative EIC chromatograms for (**A**) lovastatin standard, and in human liver microsome samples (**B**) without kinsenoside (control) and (**C**) with kinsenoside.

Because kinsenoside is considered to be an antihyperlipidemic drug candidate [2], its safety profile in relation to CYP-mediated interaction was further investigated using drugs that are most likely to be co-prescribed, such as antihypertensive and antihyperlipidemic drugs. Therefore, the most commonly prescribed drugs, amlodipine and lovastatin, were selected. Amlodipine and lovastatin were incubated concomitantly with kinsenoside in human liver microsomes under controlled conditions, in separate studies. Each reaction mixture was incubated for 1 h so that the metabolites were generated enough to obtain the clear metabolite profile. The CYP metabolites of amlodipine and lovastatin were evaluated qualitatively and compared quantitatively to determine the kinsenoside-CYP interaction. In human liver microsomes, the major metabolites identified for amlodipine were M-A1, M-A2, and M-A3, while the major metabolites for lovastatin were M-L1, M-L2, and M-L3. The parent drugs with their respective metabolites are tabulated in Table 2 and their MS/MS spectra were provided as Supplementary Materials. For quantitative analysis, the concentrations of amlodipine, lovastatin, and their CYP metabolites were calculated, and the control samples were compared with the kinsenoside-treated samples. Our findings show that kinsenoside has a negligible effect on the metabolite profile of lovastatin and amlodipine. Although a slight change (reduction) in the intensity of lovastatin peak was observed, but the metabolite peak pattern was not affected. Figure 3 and Figure 4 show the extracted ion chromatograms (EICs) for amlodipine and lovastatin with their metabolites, respectively.

## 3. Discussion

The effect of kinsenoside on CYP enzyme activities was investigated for to predict its possible interaction with CYP substrate drugs. As oral administration of kinsenoside at dose of 50 mg·kg^−1^ and 100 mg·kg^−1^ exhibited a significant antihyperlipidemic effect [2], so considering this, kinsenoside was tested in the concentration range of 0.1–100 µM, which is expected to be enough to reflect the clinically relevant concentrations. The inhibitory effect of kinsenoside on six CYP isozymes was negligible, while CYP2A6 was slightly inhibited. The extent of CYP2A6 inhibition was ~42% even at the highest concentration of kinsenoside (*i.e.*, 100 µM, Figure 2) in recombinant CYP2A6 isozyme, and the IC_50_ value was estimated greater than 100 µM. According to the FDA guideline, when [I]/Ki < 0.1, the clinical relevance of competitive CYP inhibition is predicted as “Remote”. Ki can be calculated as IC_50_/2 by assuming competitive inhibition [27]. Based on the present data, Ki would be at least 50 µM; for [I]/Ki > 0.1, plasma concentration should be higher than 500 µM. Thus, the clinically relevant inhibition is highly unlikely. Therefore, we concluded that kinsenoside showed non-significant inhibition on CYP2A6.

Kinsenoside, an antihyperlipidemic drug candidate, could possibly be used concomitantly with amlodipine and lovastatin, was evaluated further for the qualitative as well as quantitative analysis of CYP-mediated metabolites of both drugs. Lovastatin is a prescription drug frequently used to treat dyslipidemia and cardiovascular diseases and it is a specific substrate for CYP3A4 in the liver [28,29,30]. It is hydrolyzed to β-hydroxy acid lovastatin (M-L3), which is its active form *in vivo*, and then it inhibits HMG-CoA reductase and the rate-limiting step in *de novo* cholesterol synthesis [31]. It has been reported that lovastatin is metabolized by CYP3A4 in human liver microsomes to yield three major metabolites: 6-β-hydroxylovastatin (M-L1, M-L2), β-hydroxyacid lovastatin (M-L3), and 6′-exomethylene lovastatin [32,33]. In the chromatogram for lovastatin metabolism with kinsenoside, the slight decrease of the lovastatin peak was observed. This is supposed to be due to its chemical stability rather than metabolism. In details, the chemical interconversion between lactones and hydroxy acid forms are well recognized in all statin drugs and this conversion occurs in a pH-dependent manner [34]. Thus, the chemically interconvertible (pH dependent) nature of lovastatin might result in the slight reduction in lovastatin intensity. Amlodipine is a dihydropyridine calcium-channel blocker that primarily inhibits the calcium ion influx into cardiac cells and vessel smooth muscle cells, resulting in the reduction of blood pressure by peripheral arterial vasodilation [35]. It is one of the most commonly prescribed drugs for the treatment of hypertension and ischemic heart disease. Metabolite profiling data and mass balance suggested that amlodipine dehydrogenation to M-A1, followed by multiple oxidative reactions of M-A1, is the major biotransformation pathway of amlodipine in humans. It has been reported that many dihydropyridine analogs undergo CYP3A4-mediated dehydrogenation to form the corresponding pyridine metabolites *in vitro* [36]. As expected from the CYP inhibition assay results, modification for parent or major metabolites was not observed in the *in vitro* metabolism profile of amlodipine with kinsenoside.

## 4. Materials and Methods

### 4.1. Chemicals and Reagents

**Figure 5:**
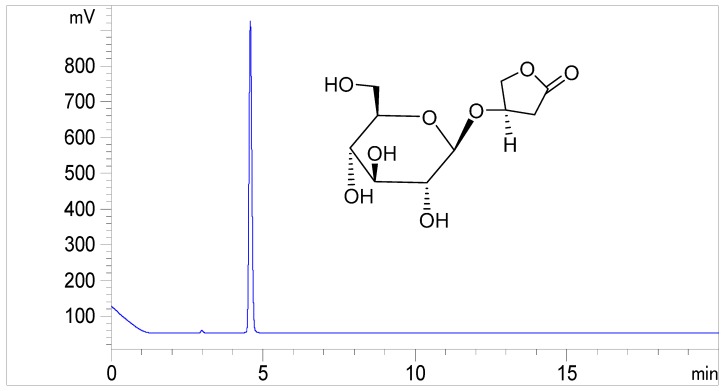
Representative HPLC-ELSD chromatogram of kinsenoside (1 µg·mL^−1^), analyzed by ELSD (evaporation temperature: 70 °C and nebulization temperature: 50 °C), with a C8 column.

Kinsenoside was contributed by Prof. Zhang, Tongji Medical College of Huazhong University of Science and Technology (Wuhan, China). Kinsenoside purity was determined by a high performance liquid chromatography with an evaporative light scattering detector (ELSD), which was >98% [37]. The HPLC-ELSD chromatogram of kinsenoside is shown in Figure 5.

Pooled human liver microsomes and recombinant CYP2A6 were purchased from BD Gentest (Woburn, MA, USA). Glucose-6-phosphate, β-NADP^+^, glucose-6-phosphate dehydrogenase, phenacetin, coumarin, diclofenac, mephenytoin, dextromethorphan, midazolam, ketoconazole, and terfenadine were obtained from Sigma Chemical Co. (St. Louis, MO, USA). Amlodipine and lovastatin were purchased from Sigma-Aldrich (St. Louis, MO, USA). All other solvents used were of HPLC grade and were purchased from J. T. Baker (Phillipsburg, NJ, USA). Distilled water was prepared using a Milli-Q purification system (Millipore, Billerica, MA, USA). All standard solutions and mobile phases were passed through a 0.22 µm membrane filter before use.

### 4.2. CYP Inhibition Assay

The reaction mixtures used in this study consisted of human liver microsomes (0.5 mg·mL^−1^), kinsenoside at different concentrations in distilled water (DW) (0.1, 0.3, 1, 3, 10, 30, and 100 µM), an NADPH-generating system (NGS) containing β-NADP^+^ (10 mg·mL^−1^), glucose-6-phosphate (0.1 M) and glucose-6-phosphate dehydrogenase (1.0 U·mL^−1^), and a mixture of substrates (phenacetin 40 µM, coumarin 2.5 µM, dextromethorphan 5 µM, diclofenac 10 µM, mephenytoin 160 µM, paclitaxel 10 µM, and midazolam 2.5 µM) in 200 µL of potassium phosphate buffer (0.05 M, pH 7.4). The reaction mixture without NGS was pre-incubated at 37 °C for 5 min and then further incubated with NGS for 30 min in a water bath. For the positive control, 5 µM ketoconazole, 50 µM sulfaphenazole, 10 µM furafylline, 10 µM methoxsalen, 30 µM quercetin, 50 µM quinidine, and 20 µM ticlopidine were tested. After incubation, the reaction was stopped by adding 400 µL of internal standard terfenadine (0.16 µM) in 0.1% acetic acid (cold ice). For the test with a recombinant CYP2A6 isozyme, cDNA-expressed CYP2A6 supersomes and a single substrate (coumarin) were used instead of human liver microsomes and the substrate mixture, respectively. The rest of the procedure was the same as described above.

### 4.3. Kinsenoside CYP Interaction Assay for Amlodipine and Lovastatin

Qualitative and quantitative analysis of CYP-mediated metabolites of amlodipine and lovastatin were conducted. In this study, the reaction mixtures consisted of human liver microsomes (0.5 mg·mL^−1^), amlodipine or lovastatin at a concentration of 10 µM, kinsenoside at a concentration of 10 µM, and an NADPH-generating system (NGS) containing β-NADP^+^ (10 mg·mL^−1^), glucose-6-phosphate (0.1 M), and glucose-6-phosphate dehydrogenase (1.0 U·mL^–1^) in 200 µL of potassium phosphate buffer (0.05 M, pH 7.4). For the control sample, kinsenoside was replaced by DW. The reaction mixture without NGS was pre-incubated at 37 °C for 5 min and then further incubated with NGS for 60 min in a water bath. After one hour, the reaction was stopped by adding 400 µL of 0.1% acetic acid (ice cold), and the sample was placed in ice until solid-phase extraction (SPE).

### 4.4. Sample Preparation

The SPE Oasis Sep-Pak C18 cartridges (HLB 96-well plate, 30 µm, Waters Co., Milford, MA, USA) were activated with 1 mL of methanol followed by 1 mL of 0.1% acetic acid in water. The incubation mixtures were loaded into the cartridge, and then washed twice with 1 mL of 0.1% acetic acid in water under vacuum. The analytes were eluted with 1 mL of methanol. The eluate was evaporated under nitrogen gas; the residue was reconstituted in 100 µL of the mobile phase. A 5 µL aliquot was injected into the respective mass spectrometry systems for analysis.

### 4.5. LC-MS/MS Analysis

An Agilent 1260 binary pump HPLC system, with the Agilent 6460 Triple Quadrupole mass spectrometer (Agilent Technologies, Palo Alto, CA, USA) equipped with an electrospray ionization (ESI) source were used as the Liquid Chromatography-Tandem Mass Spectrometry (LC-MS/MS) system. The Fortis C8 column (2.1 mm × 100 mm, 5 µm; Fortis Technologies Ltd., Cheshire, UK) was used for the chromatographic separation. The column temperature was maintained at 40 °C. The high-performance liquid chromatography (HPLC) mobile phases consisted of solvent A, 0.1% formic acid in DW and solvent B, 90% acetonitrile in solvent A. A gradient elution was used with an initial concentration of 15% of solvent B and a flow rate of 0.2 mL·min^−1^. The solvent B composition was changed as follows: 0–3.0 min, 85% (gradually increased); 3.0–4.5 min, 85% (maintained); 4.5–4.6 min, 15%; and 4.6–8.0 min, 15% (re-equilibrium). The total run time was 8.0 min, and the injection volume was 5 µL. Mass detection was performed in positive ion mode with multiple reaction monitoring (MRM). The MRM modes used for the precursor-product ion pairs (Q1/Q3) are shown in Table 3, while the representative MRM chromatograms are shown in Figure 1.

### 4.6. Liquid Chromatography/Quadrupole Time-of-Flight Mass Spectrometry (LC-QTOF MS)

For CYP metabolite analysis of amlodipine and lovastatin, an Agilent 1260 binary pump HPLC system with an Agilent 6530 LC-QTOF MS system (Agilent Technologies) was used. The chromatographic separation was performed using a Thermo Hypersil GOLD column (2.1 mm × 150 mm, 5 µm; Thermo Fisher Scientific Inc., Waltham, MA, USA), with the column temperature maintained at 40 °C. The HPLC mobile phases consisted of solvent A, 0.1% formic acid in DW and solvent B, 90% acetonitrile in solvent A. A gradient elution was used with an initial concentration of 10% of solvent B and a flow rate of 0.3 mL·min^−1^ for amlodipine. The solvent B composition was changed as follows: 0–20 min, 10%–70% (gradually increased); 20–21 min, 70%–10%; and 21–30 min, 10% (re-equilibrium). For lovastatin, the gradient elution was: initial, 30% B; 0–15 min, 30%–80%; 15–18 min, 80%; 18–18.1 min, 80%–30%; and 18.1–25 min, 30%. The injection volume was 5 µL for both amlodipine and lovastatin. A mass detection scan was performed in the positive ion mode.

## 5. Conclusions

Kinsenoside is a promising drug candidate, however, despite its pharmacological importance no study has yet shown the effects of kinsenoside on CYP enzyme activities. For the first time, this study investigated the inhibitory effect of kinsenoside on such enzymes. To predict possible drug-drug interactions, the *in vitro* metabolic profile of amlodipine (an antihypertensive drug) and lovastatin (an antihyperlipidemic drug) after treatment with kinsenoside were investigated. The results showed that there is almost no risk of potential drug interactions between kinsenoside and CYP drug substrates via CYP inhibition. Therefore, considering our findings, the drug-drug interaction between kinsenoside and other conventional drugs via CYP inhibition would be negligible.

## Figures and Tables

**Table 1:** Effects of kinsenoside on CYP-specific metabolite formation in human liver microsomes.

P450 Specific Metabolite	Metabolite Formation (% of Control)						
	Kinsenoside Concentrations (µM)						
	0.1	0.3	1	3	10	30	100
Acetaminophen (1A2)	84.3	78.8	76.4	80.1	77.5	78.3	75.6
7-OH-coumarin (2A6)	82.4	84.3	71.2	70.5	67.4	64.2	58.3
6-OH-paclitaxel (2C8)	91.8	84.2	94.4	92.6	80.5	82.9	81.6
4-OH-diclofenac (2C9)	92.2	94.5	91.7	89.2	92.5	92.3	88.5
4-OH-mephenytoin (2C19)	88.6	95.2	100.2	99.4	99.3	95.5	89.2
Dextromethorphan (2D6)	92.1	101.7	100.5	98.9	96.2	95.3	92.8
1-OH-midazolam (3A4)	88.4	91.0	94.8	89.2	85.7	86.4	82.9

**Table 2:** Major metabolite pattern of amlodipine and lovastatin in human liver microsomes.

Drug	Metabolites	RT	Chemical Formula *	Mass		Error
				Theoretical	Experimental	
Amlodipine	Parent	14.8	C_20_H_26_ClN_2_O_5_	409.1525	409.1511	3.4
	M-A1	13.1	C_20_H_24_ClN_2_O_5_	407.1368	407.1349	4.7
	M-A2	9.5	C_16_H_15_ClNO_5_	336.0633	336.0614	5.6
	M-A3	13.0	C_18_H_19_ClNO_5_	364.0946	364.0923	6.3
Lovastatin	Parent	15.8	C_24_H_36_O_5_Na	427.2455	427.2429	6.1
	M-L1	9.3	C_24_H_36_O_6_Na	443.2404	443.2403	0.4
	M-L2	9.8	C_24_H_36_O_6_Na	443.2404	443.2400	−5.6
	M-L3	13.9	C_24_H_38_O_6_Na	445.2561	445.2545	3.6

* [M + H]^+^ and [M + Na]^+^ are presented in chemical formula of amlodipine and lovastatin, respectively.

**Table 3:** Precursor-product ion pairs of CYP-specific metabolites for multiple reaction monitoring detection.

P450-Isozyme	Tested Metabolites	Precursor Ion	Product Ion
**CYP** 1A2	Acetaminophen	152.1	110.1
**CYP** 2A6	7-OH-coumarin	162.9	106.9
**CYP** 2C8	6-OH-paclitaxel	870.4	286.1
**CYP** 2C9	4-OH-diclofenac	312.2	230.9
**CYP** 2C19	4-OH-mephenytoin	235.0	150.1
**CYP** 2D6	Dextrorphan	258.3	157.1
**CYP** 3A4	1-OH-midazolam	343.1	325.1
**Internal Standard**	Terfenadine	472.4	436.4

## Supplementary Materials

Supplementary materials can be accessed at: http://www.mdpi.com/1420-3049/21/6/800/s1.

## Abbreviations

The following abbreviations are used in this manuscript:

cDNA	complementary deoxyribonucleic acid
CVD	cardiovascular disease
DW	distilled water
EIC	extracted ion chromatograms
ELSD	evaporative light scattering detector
HMG-CoA	3-hydroxy-3-methylglutaryl-coenzyme A
HPLC	high-performance liquid chromatography
LC/MS/MS	liquid chromatography/tandem mass spectrometry
M-A	amlodipine’s metabolite
M-L	lovastatin’s metabolite
NADP^+^	nicotinamide adenine dinucleotide phosphate
NGS	NADPH-generating system
STD	standard
