# Supplementary material for: In Vitro Assessment of CYP-Mediated Drug Interactions for Kinsenoside, an Antihyperlipidemic Candidate
Source: Molecules. 2016 Jun 18;21(6):800. doi: 10.3390/molecules21060800 (PMC6274256; doi:10.3390/molecules21060800)
Supplement: Supplementary file 1 [file molecules-21-00800-s001.pdf]

Supplementary Materials: *In Vitro* Assessment of CYP-Mediated Drug Interactions for Kinsenoside, an Antihyperlipidemic Candidate

Shaheed Ur Rehman, Min Sun Choi, In Sook Kim, Zengwei Luo, Yongbo Xue, Guangming Yao, Yonghui Zhang and Hye Hyun Yoo

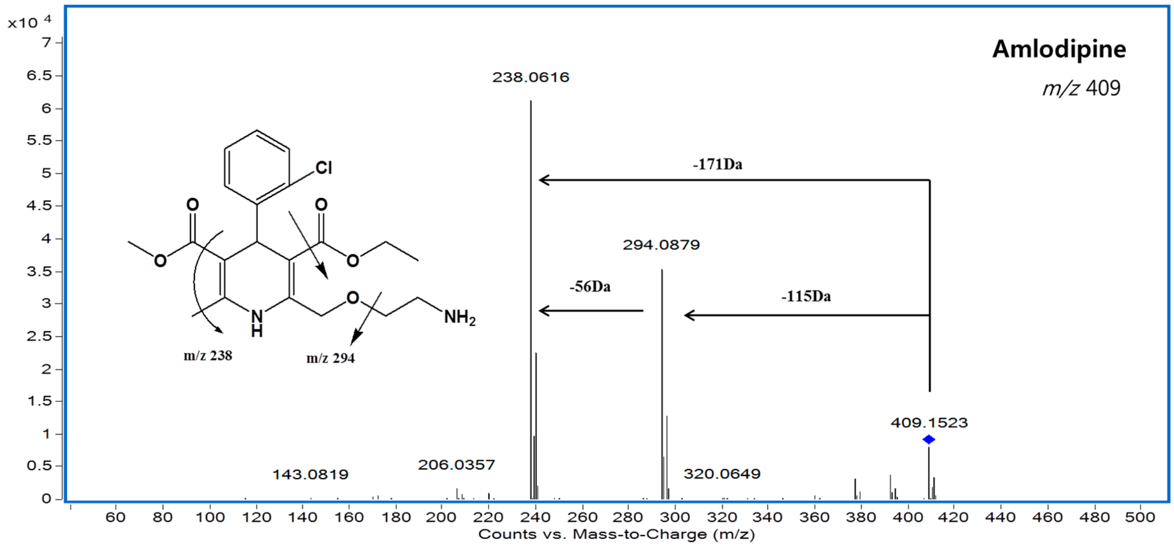

Figure S1. MS/MS spectrum of amlodipine.

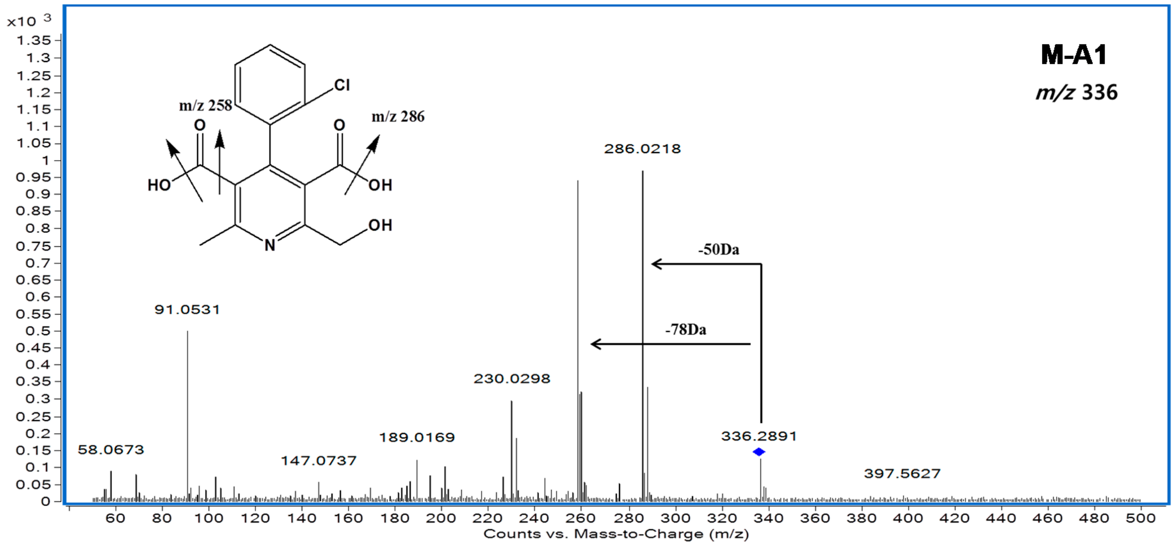

Figure S2. MS/MS spectrum of M-A1.

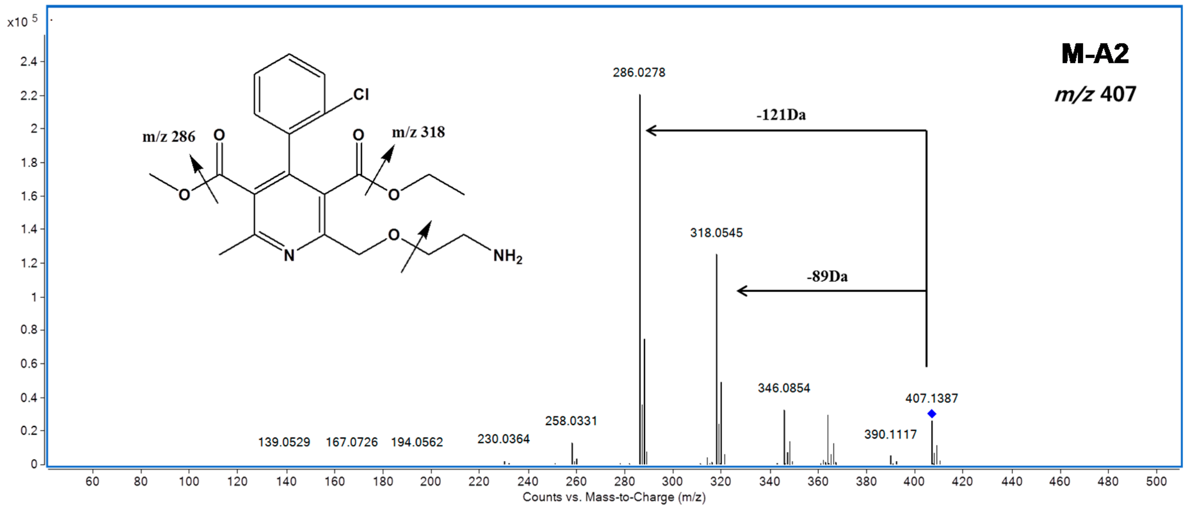

Figure S3. MS/MS spectrum of M-A2.

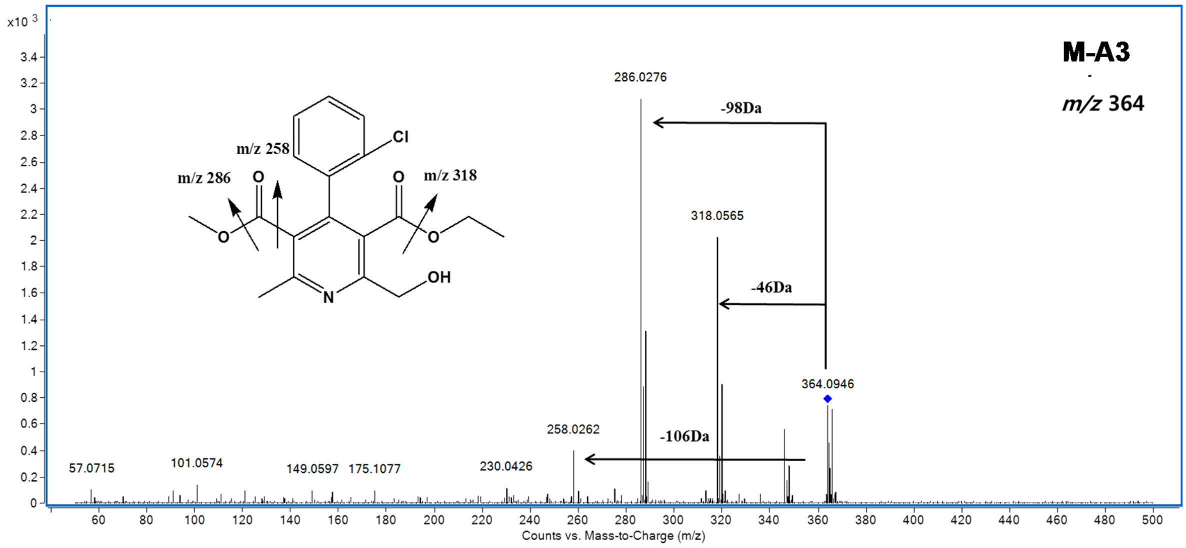

Figure S4. MS/MS spectrum of M-A3.

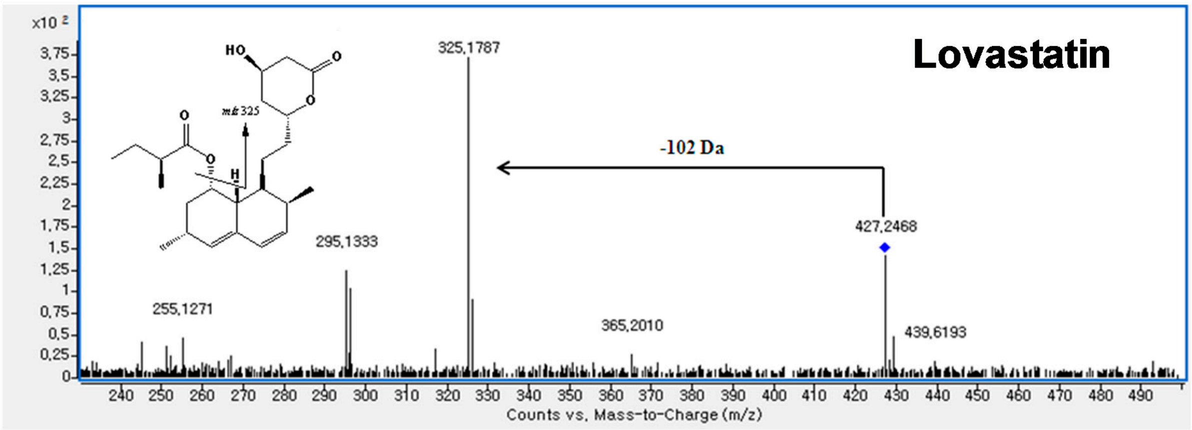

Figure S5. MS/MS spectrum of lovastatin.

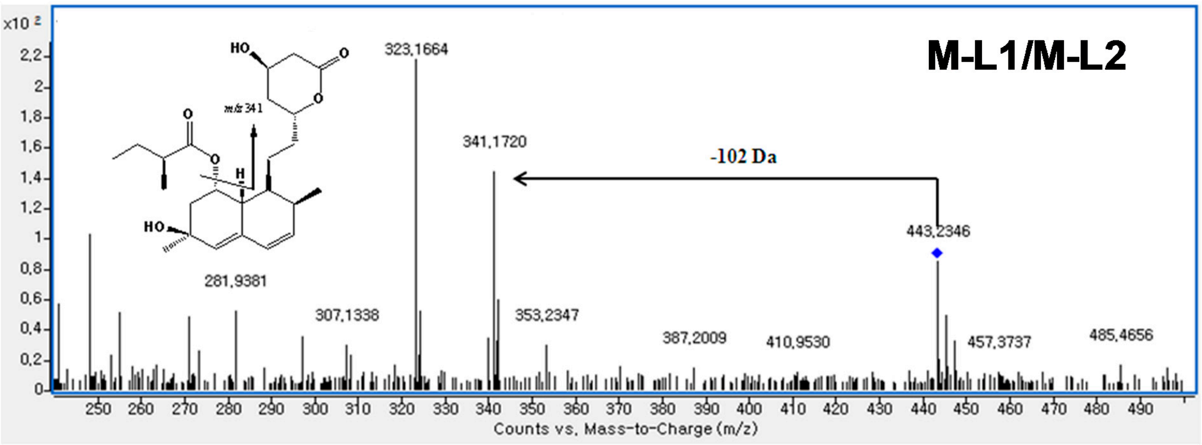

Figure S6. MS/MS spectrum of M-L1/M-L2.

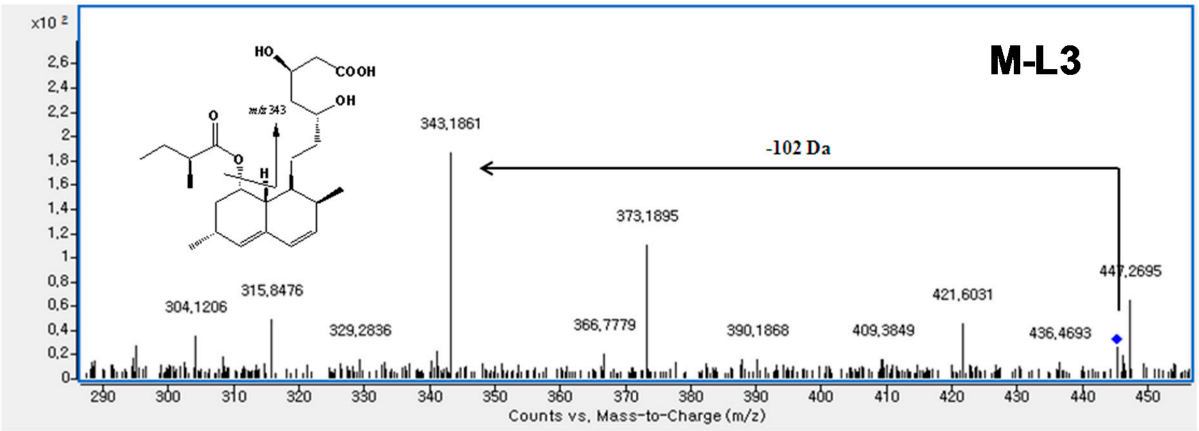

Figure S7. MS/MS spectrum of M-L3.
